# Canadian Consensus for Biomarker Testing and Treatment of TRK Fusion Cancer in Adults †

**DOI:** 10.3390/curroncol28010053

**Published:** 2021-01-15

**Authors:** D. Gwyn Bebb, Shantanu Banerji, Normand Blais, Patrice Desmeules, Sharlene Gill, Andrea Grin, Harriet Feilotter, Aaron R. Hansen, Martin Hyrcza, Monika Krzyzanowska, Barbara Melosky, Jonathan Noujaim, Bibiana Purgina, Dean Ruether, Christine E. Simmons, Denis Soulieres, Emina Emilia Torlakovic, Ming-Sound Tsao

**Affiliations:** 1 Tom Baker Cancer Centre and University of Calgary, Calgary, AB T2N 4N2, Canada; 2 Research Institute in Oncology and Hematology, CancerCare Manitoba, University of Manitoba, Winnipeg, MB R3E 0V9, Canada; sbanerji@cancercare.mb.ca; 3 Centre Hospitalier Universitaire de Montreal, Department of Medicine, University of Montreal, Montreal, QC H2X 3E4, Canada; normand.blais.chum@ssss.gouv.qc.ca (N.B.); denis.soulieres.chum@ssss.gouv.qc.ca (D.S.); 4 Service D’Anatomopathologie et de Cytologie, Institut Universitaire de Cardiologie et de Pneumologie de Québec, Université Laval, Quebec City, QC G1V 0A6, Canada; patrice.desmeules@criucpq.ulaval.ca; 5 BC Cancer, Vancouver, BC V5Z 4E6, Canada; sgill@bccancer.bc.ca (S.G.); bmelosky@bccancer.bc.ca (B.M.); christine.simmons@bccancer.bc.ca (C.E.S.); 6 Department of Pathology and Molecular Medicine, Queen’s University, Kingston, ON K7L 3N6, Canada; andrea.grin@kingstonhsc.ca (A.G.); hf4@queensu.ca (H.F.); 7 Department of Medical Oncology and Hematology, Princess Margaret Cancer Centre, University Health Network, Toronto, ON M5G 2C1, Canada; aaron.hansen@uhn.ca (A.R.H.); monika.krzyzanowska@uhn.ca (M.K.); 8 Department of Pathology and Laboratory Medicine, Arnie Charbonneau Cancer Institute, University of Calgary, Calgary, AB T2N 4Z6, Canada; martin.hyrcza@albertaprecisionlabs.ca; 9 Hôpital Maisonneuve-Rosemont, Montreal, QC H1T 2M4, Canada; john.c.njm@gmail.com; 10 The Ottawa Hospital, Department of Pathology and Laboratory Medicine, University of Ottawa, Ottawa, ON K1N 6N5, Canada; bpurgina@toh.ca; 11 Department of Oncology, Tom Baker Cancer Centre, Calgary, AB T2N 4N2, Canada; dean.ruether@albertahealthservices.ca; 12 Department of Pathology and Laboratory Medicine, Saskatchewan Health Authority and University of Saskatchewan, Saskatoon, SK S7N 5B5, Canada; emina.torlakovic@saskhealthauthority.ca; 13 Department of Pathology, Laboratory Medicine Program, University Health Network, Toronto, ON M5G 2C4, Canada

**Keywords:** NTRK, larotrectinib, entrectinib, targeted therapy, molecular testing, oncogenic drivers, TRK fusion, tumour-agnostic

## Abstract

The tyrosine receptor kinase (TRK) inhibitors larotrectinib and entrectinib were recently approved in Canada for the treatment of solid tumours harbouring neurotrophic tyrosine receptor kinase (*NTRK)* gene fusions. These *NTRK* gene fusions are oncogenic drivers found in most tumour types at a low frequency (<5%), and at a higher frequency (>80%) in a small number of rare tumours (e.g., secretory carcinoma of the salivary gland and of the breast). They are generally mutually exclusive of other common oncogenic drivers. Larotrectinib and entrectinib have demonstrated impressive overall response rates and tolerability in Phase I/II trials in patients with TRK fusion cancer with no other effective treatment options. Given the low frequency of TRK fusion cancer and the heterogeneous molecular testing landscape in Canada, identifying and optimally managing such patients represents a new challenge. We provide a Canadian consensus on when and how to test for *NTRK* gene fusions and when to consider treatment with a TRK inhibitor. We focus on five tumour types: thyroid carcinoma, colorectal carcinoma, non-small cell lung carcinoma, soft tissue sarcoma, and salivary gland carcinoma. Based on the probability of the tumour harbouring an *NTRK* gene fusion, we also suggest a tumour-agnostic consensus for *NTRK* gene fusion testing and treatment. We recommend considering a TRK inhibitor in all patients with TRK fusion cancer with no other effective treatment options.

## 1. Introduction

There are three neurotrophic tyrosine receptor kinase (*NTRK)* genes, *NTRK1, NTRK2,* and *NTRK3,* which encode the tyrosine receptor kinase (TRK) receptors, TRKA, TRKB, and TRKC, respectively [1,2,3]. In 1982, the first description of an *NTRK1* gene fusion in a colon cancer sample was published [1,4]. This was followed in 1998 by the discovery of the *ETV6-NTRK3* gene fusion in infantile fibrosarcoma (IFS) [5]. Subsequently *ETV6-NTRK3* gene fusions were found to be canonical in secretory breast carcinoma [6] and secretory carcinoma of salivary gland [7]. Under physiological conditions, TRK receptors are involved in cell differentiation, pain signalling, thermoregulation, regulation of movement, memory, mood, appetite, body weight, and proprioception [1,2,8,9,10,11,12,13].

Multiple potential 5′ partners may fuse with the 3′ end of *NTRK1-3,* resulting in constitutively activated protein, uncontrolled signaling, and oncogenesis, when the 5′ partner promotes dimerization without ligand binding [1]. These fusions generally do not co-occur with other primary oncogenic drivers [14,15].

### 1.1. Targeted Therapy for TRK Fusion Cancer

Since *NTRK* gene fusions are rare events, large randomized Phase III trials in specific tumour types are not feasible due to a long enrollment process [16,17,18]. Basket trials, where tumour-agnostic molecularly-defined groupings are used to stratify patients, have been highlighted as a necessary tool in the move toward an era of precision oncology in such situations [18]. Consequently, the TRK inhibitors larotrectinib and entrectinib have been studied in a series of basket trials, as detailed below.

Larotrectinib (VITRAKVI, Bayer Inc., Mississauga, ON, Canada) is a highly selective TRK inhibitor [16]. It is available as capsules or liquid formulation and approved at 100 mg BID in adults [19]. In pediatric patients (<18 years of age), the recommended dose is 100 mg/m^2^ BID up to a maximum of 200 mg/day [19]. It was studied in:

- Adult Phase I trial (NCT02122913) [20]
- SCOUT (pediatric {≤21 years of age} Phase I/ Phase II basket trial) (NCT02637687) [21]
- NAVIGATE (adolescent/adult {≥12 years of age} Phase II basket trial) (NCT02576431) [16].

Patients with metastatic solid tumours, regardless of *NTRK* gene fusion/alteration status, including mutations, fusions, and amplifications were included in the Phase I dose escalation trial [20]. Subsequent studies focused on the *NTRK* gene fusion-positive patient population, because drug activity was only seen in these patients [20]. A pooled analysis of these three trials is the largest and longest-term dataset for any TRK inhibitor to date [16]. At data cut-off date, 159 patients with locally advanced or metastatic TRK fusion-positive non-primary central nervous system (CNS) solid tumours and a median of one prior systemic therapy (including 107 adult patients {≥18 years of age}) were enrolled [16]. The overall response rate (ORR) (complete plus partial response) for the total population was 79%, median duration of response (DOR) was 35.2 months, median progression-free survival (PFS) was 28.3 months, and median overall survival (OS) was 44.4 months [16]. In the adults, ORR was 73% [16]. The majority of adverse events (AEs) were Grade 1–2 [16]. Eight percent of patients reduced their dose due to AEs, and 2% discontinued treatment [16]. Elevated alanine aminotransferase (two of 260 patients {<1%} who received at least one dose of larotrectinib), elevated aspartate aminotransferase (two {<1%}), and nausea (two {<1%}) were the most common larotrectinib-related serious AEs [16].

The multikinase TRK, ROS1, and ALK inhibitor entrectinib (ROZLYTREK, Hoffmann-La Roche Limited, Mississauga, ON, Canada) is approved for patients >18 years of age at 600 mg once daily and is available as capsules [22]. Health Canada has not authorized an indication for pediatric use. It was studied in the following adult trials, which included patients with locally advanced or metastatic TRK fusion-positive solid tumours [17,23]:

- ALKA-372-001 (adult Phase I basket trial) (NCT02097810)
- STARTRK-1 (adult Phase I basket trial) (NCT02097810)
- STARTRK-2 (adult Phase I basket trial) (NCT02568267)
- STARTRK-NG (adolescent/pediatric {≤20 years of age} Phase I/II basket trial) (NCT02650401)

There were 54 evaluable adult patients with TRK fusion cancer with a median of one prior systemic therapy in the pooled analysis [17]. At a data cut-off, the ORR was 57%, median DOR was 10.4 months, median PFS was 11.2 months, and median OS was 21 months [17]. Most AEs were Grade 1–2 and reversible [17]. Forty percent of patients had dose reductions, and 4% discontinued due to AEs [17]. The most common entrectinib-related serious AE was nervous system disorders (three {4%} of 68 patients with TRK fusion cancer who received at least one dose of entrectinib) [17]. Hypothetically, entrectinib may have more “off-target” effects, because it additionally inhibits ALK and ROS1.

### 1.2. Development of Resistance to TRK Inhibitors

Resistance to the first generation TRK inhibitors entrectinib and larotrectinib has been demonstrated to be the result of acquired mutations in *NTRK* that lead to mutations in the kinase domain of TRK and interfere with the binding of first-generation TRK inhibitors [24,25]. Selitrectinib, repotrectinib, and taletrectinib are second-generation TRK inhibitors, which are being studied and may overcome known resistance mechanisms [24,25,26].

### 1.3. Regulatory and Funding Status of TRK Inhibitors in Canada, as of 2020

Health Canada approved larotrectinib in July 2019 “for the treatment of adult and pediatric patients with solid tumours carrying an *NTRK* gene fusion without a known acquired resistance mutation, whose disease is metastatic, or where surgical resection is likely to result in severe morbidity, and where there are no satisfactory alternate treatment options” [19]. Health Canada approved entrectinib in February 2020 “for the treatment of adult patients with unresectable locally advanced or metastatic extracranial solid tumours, including brain metastases, that have an *NTRK* gene fusion without a known acquired resistance mutation, and with no satisfactory treatment options” [22].

### 1.4. NTRK Gene Fusions Testing

Detailed background of the methods to test for *NTRK* gene fusions is available in *Perreault* et al. published in this issue. Here, the focus is on pan-TRK immunohistochemistry (IHC), which detects TRKA, TRKB, and TRKC protein, and next generation sequencing (NGS), which detects *NTRK1, NTRK2,* and *NTRK3* gene fusions in DNA or RNA. Immunohistochemistry is recommended as a cost-efficient, widely available screening tool in some tumour types [27,28]. It has demonstrated sensitivity of 95.2% and specificity of 100% in predominantly non-CNS tumour types, 82% in breast carcinoma, and 53% in salivary gland carcinoma, with poor sensitivity and specify in sarcoma [29,30]. In IHC-positive cases, the diagnosis of TRK fusion cancer must be confirmed with a molecular test, such as NGS, because IHC detects protein expression, which could come from either wildtype or fusion TRK protein. In turn, NGS can simultaneously assess multiple mutations/fusions using multigene panels and has a high sensitivity and specificity compared to IHC [31,32].

To address potential shortfalls, these methods will be validated in the CANTRK Ring Study, a Canadian multi-centre *NTRK* gene fusion validation study, which aims to harmonize and standardize Canadian molecular pathology laboratory approaches to testing [33]. The CANTRK Ring Study was designed to assist laboratories across the country to validate laboratory-developed IHC assays for pan-TRK screening as well as comprehensive molecular testing by NGS to detect *NTRK* gene fusions, relying on existing diagnostic laboratory infrastructure [33].

## 2. Method to Achieve Consensus on TRK Fusion Cancer Algorithms

In early 2018, a group of Canadian experts including medical oncologists, endocrinologists, pathologists, and molecular laboratory directors was assembled. Between 2018–2019, a series of consultancy meetings were held, during which the authors developed draft algorithms for testing and treatment of TRK fusion cancer. These tumour-specific algorithms were then used as the basis for discussion and further refinement. Consensus on these algorithms was reached through a series of teleconferences and emails. The algorithms and draft text were subsequently revised and recirculated through an iterative process until all authors agreed with and signed off on the final content.

While this publication focuses on five tumour types (thyroid carcinoma, colorectal carcinoma {CRC}, non-small cell lung carcinoma {NSCLC}, soft tissue sarcoma {STS}, and salivary gland carcinoma), *NTRK* gene fusions have been identified in over 20 different tumour types. There is a high unmet need for testing to identify locally advanced or metastatic patients with TRK fusion cancer in all solid tumour types [31]. Therefore, we aim to provide a Canadian consensus on how to identify and treat patients with TRK fusion cancer. Evidence to support estimated cases per year in Canada and proportions of patients who might receive *NTRK* gene fusion testing is included either from the literature where possible or from clinical experience gained through Canadian efforts to establish an *NTRK* testing program.

## 3. Thyroid Carcinoma

### 3.1. Background

The Canadian Cancer Society estimates there will be 8600 new cases of thyroid carcinoma in 2020 [34]. The majority (90%) of cases are well differentiated thyroid carcinoma (DTC; papillary and follicular), and the remaining are either medullary (MTC; 2–3%) or anaplastic and poorly differentiated (ATC; 7–8%) [35]. Differentiated thyroid carcinoma and MTC are somewhat unique from the other tumour types discussed, in that, for cases not cured by surgery +/− radioactive iodine (RAI), the typical disease course progresses slowly [36]. In contrast, ATC progresses very rapidly and is often unresectable at presentation [36]. In a series of 11 cases from a single institution (10 papillary thyroid carcinomas and one secretory carcinoma), TRK fusion-positive thyroid carcinomas were clinically aggressive with high metastatic rate, multinodular growth, and lymphovascular spread [37].

There may be a misperception that there is no need for new therapies in thyroid carcinoma, because ≥85% of patients with DTC are cured [38]. However, there is a subpopulation with persistent, RAI-refractory disease that may be highly symptomatic, with poor prognosis, and limited treatment options. These patients would benefit from a targeted therapy option with a good tolerability profile.

The 2019 European Society of Medical Oncology (ESMO) guidelines recommend lenvatinib or sorafenib for RAI-refractory patients with DTC [36]. Currently, sorafenib is not publicly funded in Canada [39]. In the Phase III SELECT trial, patients with progressive RAI-refractory disease who received lenvatinib had a response rate of 64.8% and 14.2% of patients who discontinued lenvatinib due to toxicity [40]. Common practice second-line in Canada is an alternative multi-kinase inhibitor (sorafenib, sunitinib, or cabozantinib), followed by a clinical trial. The ESMO guidelines recommend cabozantinib or vandetanib first-line or second-line for MTC, although cabozantinib is not publicly funded in Canada [36]. In the Phase III EXAM trial, cabozantinib demonstrated an ORR of 28%, and in the Phase III ZETA trial, vandetanib demonstrated an ORR of 45% [41,42]. The ESMO guidelines recommend cytotoxic chemotherapy first-line for wildtype ATC, although response rates are poor [36]. There is no evidence to support second-line decisions in ATC [36].

The 26 patients with TRK fusion-positive thyroid carcinoma (19 papillary thyroid cancer, five ATC, and two follicular thyroid cancer) treated with larotrectinib in the adult Phase I and the NAVIGATE trial (which included two pediatric patients) had an ORR of 79% with median DOR not estimable [16,43]. None of the patients discontinued treatment due to AEs [16,43]. In the Phase I/II entrectinib trials, of the five patients with TRK fusion-positive thyroid carcinoma (histology not reported), one had a response, and the DOR was not reported [17].

### 3.2. Testing Consensus

**Figure 1:**
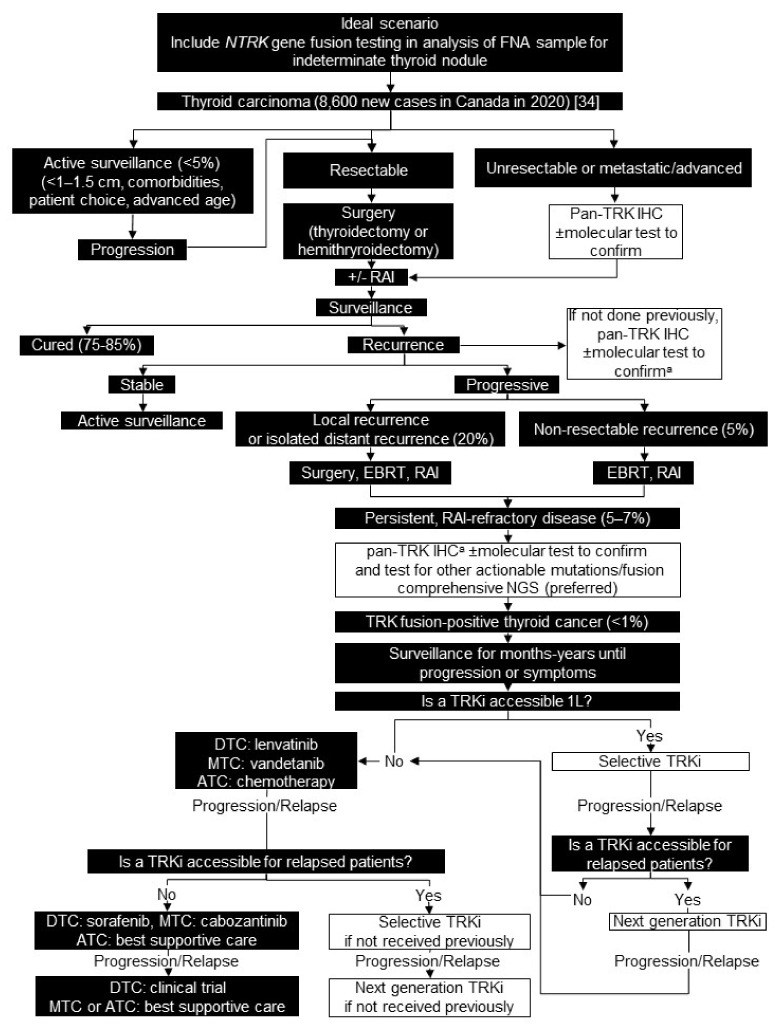
Biomarker testing and treatment for neurotrophic tyrosine receptor kinase (*NTRK*) gene fusions in thyroid carcinoma. White boxes with black outlines represent either *NTRK* gene fusion testing or treatment with a TRKi. Black boxes with white text indicate all other steps that do not include either *NTRK* gene fusion testing or treatment with a TRKi. ^a^ Consider obtaining fresh tissue to confirm diagnosis and provide more recent tissue for biomarker testing. ATC = anaplastic thyroid carcinoma; DTC = differentiated thyroid carcinoma; EBRT = external beam radiation therapy; FNA = fine needle aspiration; IHC = immunohistochemistry; L = line; MTC = medullary thyroid carcinoma; NGS = next generation sequencing; RAI = radioactive iodine; TRK = tyrosine receptor kinase; TRKi = TRK inhibitor. Brenner et al. 2020 [34].

We recommend routine testing for *NTRK1-3* gene fusions at diagnosis for unresectable or metastatic/advanced patients for all thyroid histologies. For patients with resectable disease, we recommend testing at recurrence after surgery with or without RAI, and for all patients with RAI-refractory disease if not already performed (Figure 1).

We recommend testing all patients using pan-TRK IHC, followed by comprehensive molecular testing for *NTRK1-3* gene fusions by NGS to confirm IHC-positive or equivocal cases. Some institutions perform biomarker testing on fine needle aspiration samples from indeterminate thyroid nodules for diagnostic purposes. Ideally, testing for *NTRK1-3* gene fusions could be included in this panel to identify patients as early as possible.

### 3.3. Treatment Consensus

Systemic therapy should be considered for patients with persistent RAI-refractory thyroid carcinoma. We recommend a selective TRK inhibitor be considered, if accessible, for all patients with TRK fusion-positive thyroid carcinoma as the first systemic treatment, because it is generally well tolerated and has a comparable response rate to standard of care (Figure 1).

## 4. Colorectal Carcinoma

### 4.1. Background

There will be an estimated 26,900 new cases of CRC in Canada in 2020 [34]. In two retrospective analyses of 4569 and 7008 CRC tumours, the frequency of TRK fusion-positive CRC was 0.2% [44,45].

For patients with metastatic CRC who are eligible for first-line therapy (estimated 75% of patients diagnosed each year), tumour IHC testing for mismatch repair deficiency (dMMR) and NGS testing for extended *RAS* and *BRAF* mutations is the standard clinical practice. While it would be ideal for *NTRK1-3* gene fusions testing to be incorporated into this routine panel, this is currently not cost-effective or feasible at many centres.

Approximately 10–20% of CRCs are microsatellite instability (MSI)-high [46,47,48,49,50]. In a retrospective study, MSI-high/dMMR patients with CRC and oncogenic fusions had a 40% three-year cancer-specific survival vs. 97% in MSI-high/dMMR patients without oncogenic fusions, highlighting the need for effective therapies in the former group [51]. Across several trials, 76.9–89% of patients with TRK fusion-positive CRC were also dMMR/MSI-high [15,44,52,53,54]. Generally, *NTRK* gene fusions are also mutually exclusive with other primary oncogenic drivers, such as *RAS* family genes and *BRAF* mutations [44,53]. It is therefore possible to use an exclusionary testing approach to screen for *NTRK* gene fusions in MSI-high/dMMR and *BRAF* V600E wild-type CRC tumours [44], and recent literature supports that 15% of these CRC tumours harbour a kinase fusion, including *NTRK* fusions [54].

According to a Canadian consensus statement, FOLFOX/FOLFIRI (folinic acid, fluorouracil and oxaliplatin/irinotecan) + bevacizumab is standard of care for patients with *RAS* wildtype right-sided primary tumours [55]. In the Phase III CALGB 80405 trial, patients with right-sided primary tumours treated with first-line chemotherapy + bevacizumab demonstrated an ORR of 39.7% [56]. The Canadian consensus recommends chemotherapy in combination with an anti-EGFR monoclonal antibody (cetuximab or panitumumab) in patients with *RAS* wildtype left-sided tumours [55]. In the CALGB 80405 trial, patients with left-sided primary tumours treated with first-line chemotherapy + anti-EGFR monoclonal antibody demonstrated an ORR of 69.4%.

Pembrolizumab is currently approved in Canada as monotherapy for patients with MSI/dMMR CRC following a fluoropyrimidine, oxaliplatin, and irinotecan [57]. In future, pembrolizumab monotherapy may also be an option for patients with MSI-high/dMMR tumours first-line, which demonstrated an ORR of 43.8% and a median DOR not reached, with a median follow-up of 28.4 months [58].

Second-line therapy depends on MMR/MSI status. In the Phase II KEYNOTE-164 trial, patients with CRC who had received ≥2 prior lines of therapy, were MSI-high/dMMR, and received pembrolizumab had an ORR of 33% [59]. For proficient MMR patients (pMMR), not eligible for second-line immunotherapy, the Canadian consensus recommends standard chemotherapy [55]. Bevacizumab would be included for any patients who did not receive it first-line [55].

The eight adult patients with TRK fusion-positive CRC treated with larotrectinib in the Phase I/II clinical trials had an ORR of 50% and a median DOR of 3.7 months [16,52]. The majority of patients had received one or more prior systemic therapies [16,52]. One of the four patients (25%) with TRK fusion-positive CRC treated with entrectinib in the Phase I/II trials had a response, and the DOR was not reported [17].

### 4.2. Testing Consensus

**Figure 2:**
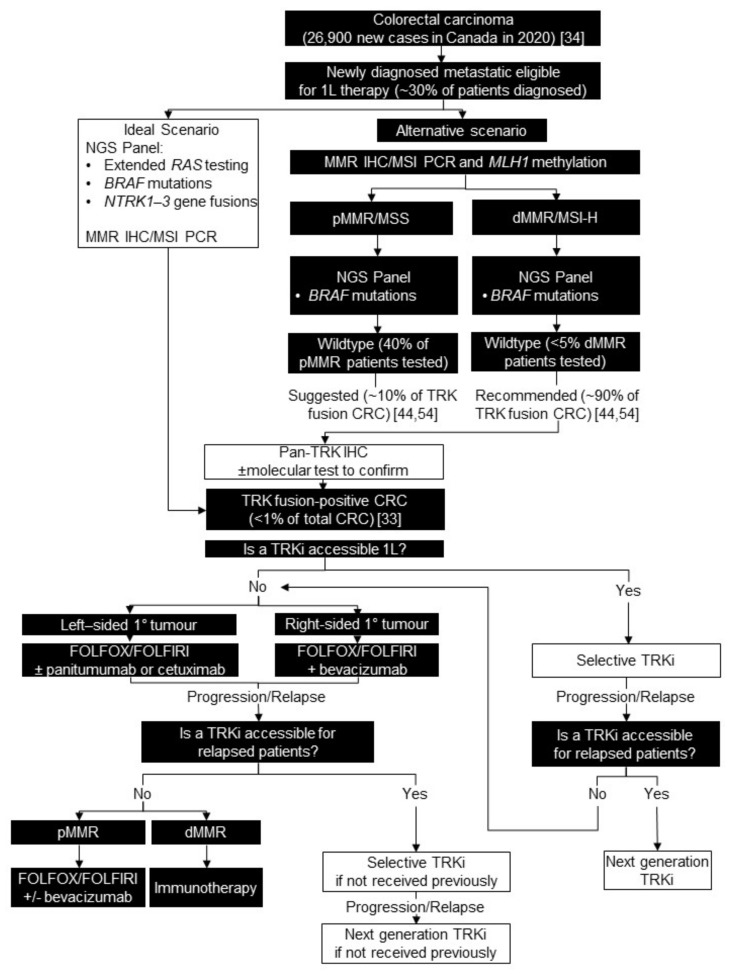
Biomarker testing and treatment for neurotrophic tyrosine receptor kinase (*NTRK)* gene fusions in colorectal carcinoma. White boxes with black outlines represent either *NTRK* gene fusion testing or treatment with a TRKi. Black boxes with white text indicate all other steps that do not include either NTRK gene fusion testing or treatment with a TRKi. *BRAF* = B-Raf proto-oncogene, serine/threonine kinase; CRC = colorectal carcinoma; d = deficient; FOLFIRI = folinic acid, fluorouracil, and irinotecan; FOLFOX = folinic acid, fluorouracil, and oxaliplatin; IHC = immunohistochemistry; L = line; *MLH1* = mutL homolog 1; MMR = mismatch repair; MSI = microsatellite instability; MSI-H = MSI high; MSS = microsatellite sable; NGS = next generation sequencing; p = proficient; PCR = polymerase chain reaction; *RAS* = RAS type GTPase family; TRK = tyrosine receptor kinase; TRKi = TRK inhibitor. Brenner et al. 2020 [34]; Chou et al. 2019 [44]; Cocco et al. 2019 [54].

We recommend reflex testing for *NTRK* gene fusions in all *RAS/BRAF* wildtype, MSI-high/dMMR CRC tumours. We estimate this strategy would result in <5% of metastatic patients requiring *NTRK1-3* gene fusion testing. In a retrospective analysis, the frequency of TRK fusion-positive CRC was 5.3% in this population [44]. Given that 11–23.1% of TRK fusion-positive CRC has been identified in microsatellite stable/pMMR, it is reasonable to also consider *NTRK* gene fusion testing in microsatellite stable/pMMR, *RAS/BRAF* wildtype patients (~40% of metastatic patients) if resources permit (Figure 2) [15,44,52,53,54].

In two studies, 86% and 89% of patients with TRK fusion-positive CRC were deficient in *MLH1*/ *PMS2* as a result of *MLH1* promoter hypermethylation [44,54]. In the future, as more data becomes available, it may be recommended to narrow the testing eligibility further to *RAS/BRAF* wildtype mCRC with *MLH1* promoter hypermethylation.

### 4.3. Treatment Consensus

Currently, selective TRK inhibitors are indicated for patients for whom alternative satisfactory options do not exist. In a retrospective chart review of Canadian patients with CRC, only 70% received second-line therapy [60]. Recognizing the risk of treatment attrition with sequential lines of therapy, it may be reasonable to consider TRK inhibitors in the first-line setting. This is a rare molecularly selected metastatic CRC patient population who may not be able to access post-progression therapy with a TRK inhibitor if there is a subsequent decline in their functional and performance status (Figure 2).

## 5. Non-Small Cell Lung Cancer

### 5.1. Background

There will be an estimated 29,800 new cases of lung cancer in Canada in 2020 [34]. Based on current data, NSCLC has a low probability of harbouring an *NTRK* gene fusion, estimated at under 0.23% [61]. However, *NTRK* gene fusions are more common in younger patients (median age at diagnosis 47.6 years), patients with a 0–5 pack–year smoking history (73% of patients with *NTRK* gene fusions), and patients with adenocarcinoma (82% of cases of patients with *NTRK* gene fusions), although they were found across gender, age, histology, stage at diagnosis, and smoking history [61].

Pembrolizumab monotherapy is approved for all patients with NSCLC with ≥1% PD-L1 tumour proportion score (TPS) without an EGFR or ALK aberration [57]. Pembrolizumab in combination with chemotherapy is approved by Health Canada as first-line systemic treatment for advanced NSCLC regardless of TPS [57]. According to the joint American Society of Clinical Oncology and Ontario Health guidelines, the standard of care for advanced stage NSCLC patients with ≥50% PD-L1 TPS is pembrolizumab monotherapy, unless a targetable oncogenic driver mutation is identified [62]. In patients with high symptom/disease burden, large volume of visceral tumour involvement, or if the treating clinician deems appropriate, pembrolizumab with chemotherapy may be given [62]. For patients with a TPS <50%, pembrolizumab/carboplatin/pemetrexed (non-squamous) or pembrolizumab with suitable platinum doublet (squamous) are recommended [62]. These standard of care options have demonstrated ORRs in clinical trials of 44.8%, 49.2%, and 49.5%, respectively [63,64,65]. Second- and third-line options include nivolumab, pembrolizumab, atezolizumab, docetaxel, vinorelbine, or pemetrexed (depending on histology and previous treatment) [66].

For *EGFR* activating mutation-positive tumours or *ALK/ROS1* fusion-positive tumours, the high response rate (43–80% for *EGFR* tyrosine kinase inhibitor {TKIs} [67,68,69,70,71] and 60–92% for ALK TKIs [72,73,74,75,76]), increased survival, and excellent tolerability of oral TKIs make these the agents of choice [77]. When a tumour is found to harbour a driver mutation as well as being PD-L1 high, treatment with the appropriate TKI is recommended [66].

In a pooled analysis of the three Phase I/II larotrectinib clinical trials, the 12 adult patients with TRK fusion-positive NSCLC demonstrated an ORR of 75% with median DOR not estimable [16,78]. The ORR was 60% in five evaluable patients with TRK fusion-positive solid tumours with brain metastases treated with larotrectinib (four out of six enrolled patients had NSCLC), demonstrating larotrectinib is active in patients with intracranial disease [79]. In an integrated analysis of the Phase I/II entrectinib trials, 10 patients with TRK fusion-positive NSCLC demonstrated an ORR of 70%, and the median DOR was not reported [80].

### 5.2. Testing Consensus

Widespread use of comprehensive NGS testing across institutions is currently challenging. It would be acceptable to screen non-squamous patients with a molecular test for recognized actionable mutations/indels, with simultaneous IHC screening for ALK, ROS1, pan-TRK, and PD-L1 protein expression. Refer to the College of American Pathologists/International Association for the Study of Lung Cancer/Association for Molecular Pathology Clinical Practice Guideline for recommendations on molecular testing [81]. The target for clinical utility of the IHC laboratory developed test screening assays for ALK, ROS1, and pan-TRK should aim at 100% sensitivity, 100% negative predictive value, and >90% positive predictive value. Confirmation of gene fusions is required for ROS1- and pan-TRK-IHC positive cases, ideally with NGS, but minimally with FISH or another validated method. If necessary, to enrich the population for testing further, some institutions may consider sequential reflex testing of patients who are *EGFR-, ALK-, ROS1-, NRG1-,* and *RET*-wildtype (~65% of non-squamous NSCLC), as *NTRK* gene fusions are considered mutually exclusive with these other primary oncogenic drivers.

**Figure 3:**
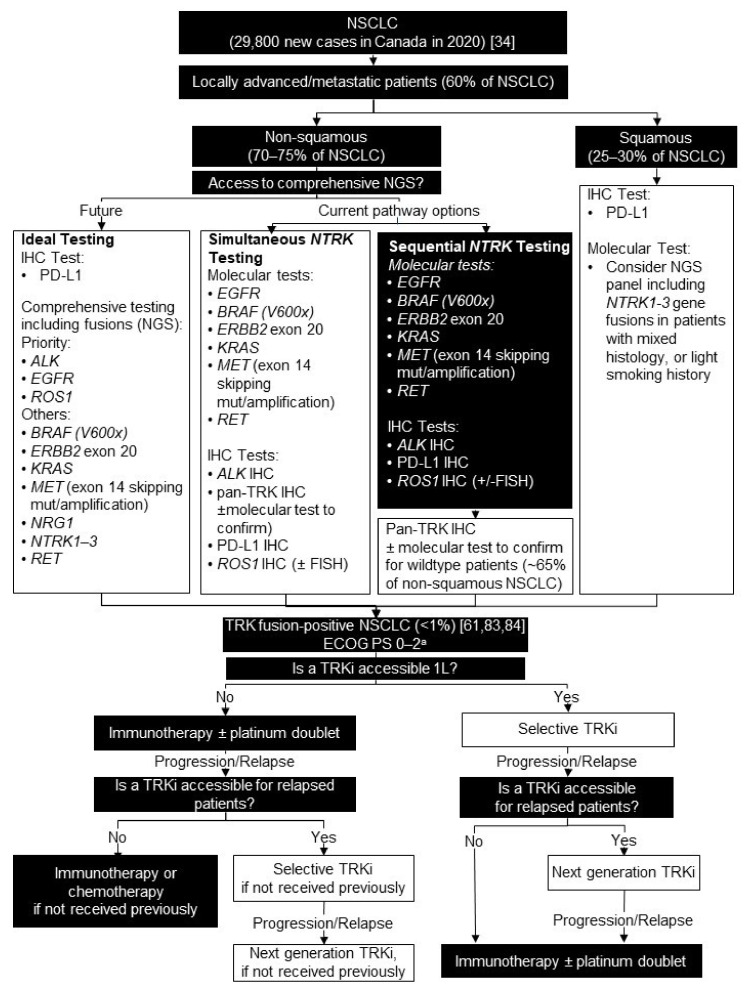
Biomarker testing and treatment for neurotrophic tyrosine receptor kinase (*NTRK)* gene fusions in lung cancer. White boxes with black outlines represent either *NTRK* gene fusion testing or treatment with a TRKi. Black boxes with white text indicate all other steps that do not include either NTRK gene fusion testing or treatment with a TRKi. ^a^ Patients with ECOG PS 3 were eligible for enrollment in the larotrectinib clinical trials [16]. *ALK* = ALK receptor tyrosine kinase; *BRAF* = B-Raf proto-oncogene, serine/threonine kinase; ECOG PS = Eastern Cooperative Oncology Group performance status; *EGFR* = epidermal growth factor receptor; *ERBB2* = erb-b2 receptor tyrosine kinase 2; FISH = fluorescence in situ hybridization; IHC = immunohistochemistry; *KRAS* = KRAS proto-oncogene, GTPase; L = line; *MET* = MET proto-oncogene, receptor tyrosine kinase; NGS = next generation sequencing; *NRG1* = neuregulin 1; NSCLC = non-small cell lung carcinoma; PD-L1 = programmed death ligand 1; *RET* = ret proto-oncogene; *ROS1* = ROS proto-oncogene 1, receptor tyrosine kinase; TRK = tyrosine receptor kinase; TRKi = TRK inhibitor. Brenner et al. 2020 [34]; Farago et al. 2018 [61]; Solomon et al. 2019 [83]; Benayed et al. 2019 [84].

We recommend integration of routine testing for *NTRK1-3* gene fusions in Stage III/IV patients with locally advanced or metastatic disease (we estimate this would include 60% of patients with NSCLC/year in Canada) [82]. Ideally, non-squamous patients (estimated at 70–75% of locally advanced/metastatic patients/year in Canada) would receive IHC testing for PD-L1 and molecular testing including *EGFR* mutations; gene fusions involving *ALK*, *ROS1*, *NRG1, NTRK1-3*, and *RET*; and other genes with known oncogenic drivers (e.g., *BRAF (V600x)*, *ERBB2 exon 20, KRAS,* and *MET* (exon 14 skipping, mutation, or amplification)) (Figure 3). Comprehensive molecular testing should be considered for selected patients with squamous cell carcinoma with mixed histology or light smoking history.

### 5.3. Treatment Consensus

Similar to *EGFR/ALK/ROS1* mutation-positive lung cancer, we recommend a selective TRK inhibitor first-line, if accessible for TRK fusion-positive lung cancer. This is based on the totality of evidence in patients with TRK fusion cancer, the fact *NTRK* gene fusions are a primary oncogenic driver, and the impressive ORR demonstrated in patients with TRK fusion-positive lung cancer compared with standards of care. Should patients develop resistance to larotrectinib or entrectinib, we recommend consideration of a clinical trial. Selitrectinib and repotrectinib are next generation TRK inhibitors, which are currently in development to overcome resistance [77]. Subsequently, standard cytotoxic chemotherapy can be offered to those of adequate performance status (Figure 3).

## 6. Soft Tissue Sarcoma

### 6.1. Background

Sarcomas are a heterogeneous group of tumours arising from bone, muscle, fat, or connective tissue, representing <1–3% of all adult cancers [85,86]. Over 70 different sub-types have been documented, each characterized by a unique and distinct biology [87,88,89]. Sarcomas can be classified into two major groups, consisting of bone sarcomas (~20%) and STSs (~80%) [90,91]. In 2016, 240 Canadians were diagnosed with bone tumours, and 1025 Canadians were diagnosed with STS [90,91].

According to a recent review of TRK fusion-positive mesenchymal tumours, the morphological characteristics indicative of a higher probability of harbouring an *NTRK* gene fusion were inflammatory myofibroblastic tumour-like, fibrosarcoma/malignant peripheral nerve sheath tumour-like, or lipofibromatosis-like morphological patterns, particularly if combined with S100 and CD34 co-expression [92].

In adult gastrointestinal stromal tumours (GISTs) (18% of STS) [88], *NTRK* gene fusions are considered mutually exclusive with other oncogenic drivers [15]. The majority of GISTs harbour activating *KIT* or *PDGFRA* mutations [93]. However, 15% of cases are *KIT/PDGFRA* wildtype, and most are associated with deficiency in the succinate dehydrogenase (SDH) enzyme or a *BRAF* mutation [93,94,95,96,97,98,99]. Some cases of *KIT/PDGFRA* wildtype tumours have been shown to harbour an *NTRK* gene fusion [100]. Whether these are true GISTs or rather a completely distinct group of gastrointestinal mesenchymal tumours will need to be studied further [100]. Patients with wildtype GIST are less responsive to imatinib and may be less responsive to sunitinib and regorafenib (the conventional treatments for *KIT/PDGFRA* mutated GIST) [93,101], and thus there is an urgent need for effective treatments. 

For non-GIST STS (82% of STS) [88], to the best of our knowledge, TRK-fusion positive STSs are mutually exclusive with other known fusion-positive sarcomas [15], which represent roughly a third of cases [88]. In addition, no *NTRK* gene fusions have been documented to date in leiomyosarcoma and liposarcoma subtypes, consisting of roughly another third of cases [88].

According to a Canadian review of systemic therapy for advanced STS, doxorubicin is the agent of choice, either alone or in combination with ifosfamide, which improves the response rate but not the OS [87]. The large number of STS subtypes makes it challenging to assess the relative efficacy of systemic therapies, because of the heterogeneous chemosensitivity of subtypes in the clinical trial populations [87]. Single agent doxorubicin has demonstrated response rates of 11.9–27% compared to combination doxorubicin ± ifosfamide, which has slightly higher response rates of 26–45.9% [87,102,103,104,105,106,107,108,109]. In subsequent treatment lines, the Canadian review recommends doxorubicin (if not used first-line), gemcitabine-docetaxel, eribulin (for liposarcoma), single agents (dacarbazine, gemcitabine, pazopanib, or trabectedin), or a clinical trial [87]. However, ORRs are low, and the clinical benefit is limited [87].

Twenty-three adult patients in the Phase I/II larotrectinib trials had TRK fusion-positive sarcoma [16,110]. The response rate was 74%, and median DOR for the entire population (which included 48 pediatric patients with TRK-fusion positive sarcoma) was not estimable [16,110]. There were 13 patients with STS in the entrectinib Phase I/II trials who had an ORR of 46%, while median DOR was not reported [17].

### 6.2. Testing Consensus

Many sarcomas have pathognomonic genomic aberrations that are best detected by NGS. For patients with *KIT/PDGFRA* wildtype GIST, we recommend testing for *NTRK1-3* gene fusions and *BRAF* mutations using NGS. We suggest testing all patients with NGS without a pan-TRK IHC screen, because pan-TRK IHC screening is associated with faint staining in GIST and can be challenging to interpret, and the volume of patients in this category would be low [111].

**Figure 4:**
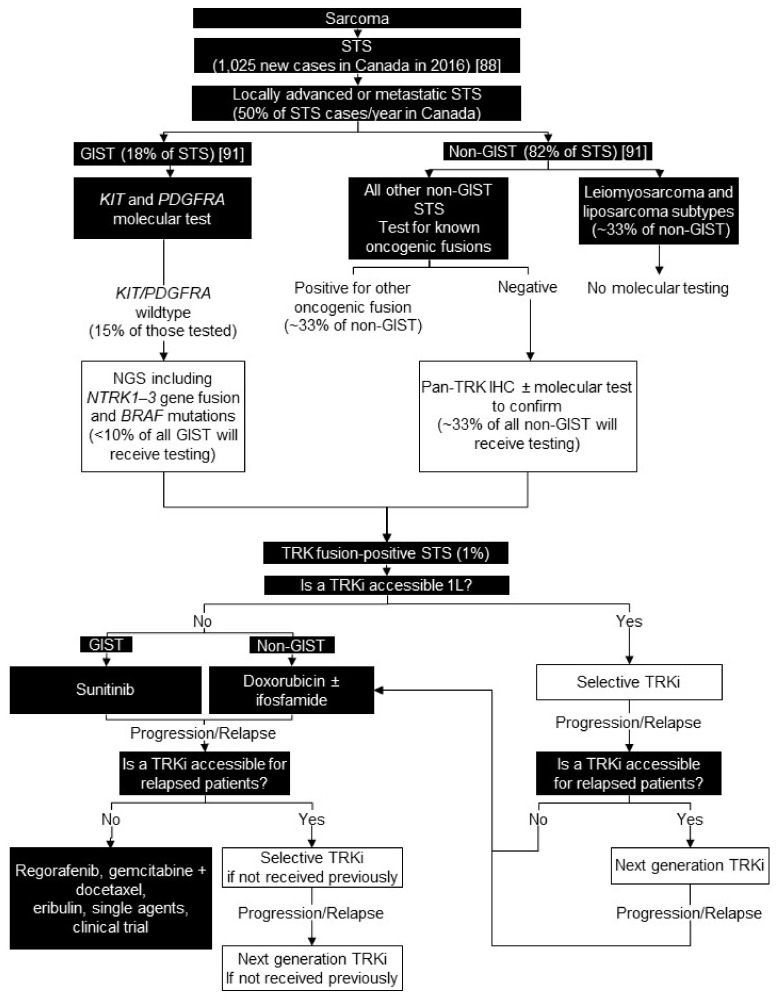
Biomarker testing and treatment for neurotrophic tyrosine receptor kinase (*NTRK)* gene fusions in sarcoma. White boxes with black outlines represent either *NTRK* gene fusion testing or treatment with a TRKi. Black boxes with white text indicate all other steps that do not include either NTRK gene fusion testing or treatment with a TRKi. *BRAF* = B-Raf proto-oncogene, serine/threonine kinase; GIST = gastrointestinal stromal tumour; IHC = immunohistochemistry; *KIT* = KIT proto-oncogene, receptor tyrosine kinase; L = line; NGS = next generation sequencing; *PDGFRA* = platelet derived growth factor receptor alpha; STS = soft tissue sarcoma; TRK = tyrosine receptor kinase; TRKi = TRK inhibitor. Ducimetière et al. 2011 [88]; Canadian Cancer Society [91].

We recommend *NTRK* gene fusion testing of all other sarcoma subtypes in locally advanced or metastatic patients using pan-TRK IHC, with confirmatory NGS on IHC-positive or IHC-inconclusive cases (Figure 4).

### 6.3. Treatment Consensus

We recommend a selective TRK inhibitor as the first-line systemic treatment in TRK fusion- positive GISTs and other TRK fusion-positive STS because of the impressive response rates, DOR, and better tolerance compared with standard of care options (Figure 4).

## 7. Salivary Gland Tumours

### 7.1. Background

The most recent incidence statistics for salivary gland carcinomas are from 2016, when 430 Canadians were diagnosed [112].

Secretory carcinomas of the salivary gland were first described in 2010 and make up approximately 4–5% of salivary gland carcinomas [7,113,114]. The majority of secretory carcinomas have *NTRK3* gene fusions (most often *ETV6-NTRK3)* [7], but there are reports of other *ETV6* fusion partners, such as *RET*, *MET,* and *MAML3* [115,116,117,118].

Salivary ductal carcinoma and adenocarcinoma not otherwise specified (estimated 70% of unresectable/metastatic/incurable salivary gland carcinoma) are routinely tested using IHC for androgen receptor expression and *HER2* expression [119].

To date, there is no evidence that chemotherapy prolongs survival in patients with metastatic salivary gland carcinoma, so the goal is palliation. For patients with *HER2*-negative and androgen receptor-negative disease, the National Comprehensive Cancer Network guidelines recommend cisplatin/vinorelbine or cisplatin/doxorubicin/cyclophosphamide [120]. Platinum-containing combination chemotherapy regimens are typically used and have demonstrated an ORR of ~25–30% in clinical trials [121,122]. Chemotherapy is also used second-line, except for patients with adenoid cystic carcinoma, where VEGFR TKIs such as lenvatinib have shown modest activity and can be considered on a case-by-case basis [123].

Of the 20 patients with TRK fusion-positive salivary gland tumours treated with larotrectinib in the Phase I/II trials, there was a 90% ORR and a 35.2 month median DOR [16]. In the entrectinib Phase I/II trials, among the seven patients with TRK fusion-positive secretory carcinoma, there was an 86% ORR and median DOR for the patients with secretory carcinoma was not reported [17].

### 7.2. Testing Consensus

For secretory carcinoma of the salivary gland, we recommend NGS for *NTRK1-3* and *RET* gene fusions. For *RET* fusions, they are actionable in the minority of secretory carcinoma cases that have them. For all other salivary gland carcinomas, we recommend molecular profiling in locally advanced, incurable, or recurrent metastatic patients, to determine eligibility for therapy or clinical trials, depending on histology. As part of this deep molecular tumour interrogation, we recommend simultaneous routine molecular testing for *NTRK1-3* gene fusions. Alternatively, some institutions may choose to reflexively test only patients who are androgen receptor-negative and *HER2*-negative, because *NTRK* gene fusions are considered mutually exclusive with other primary oncogenic drivers [15]. We estimate this strategy would reduce the number of patients with these histologies to test by >90%.

**Figure 5:**
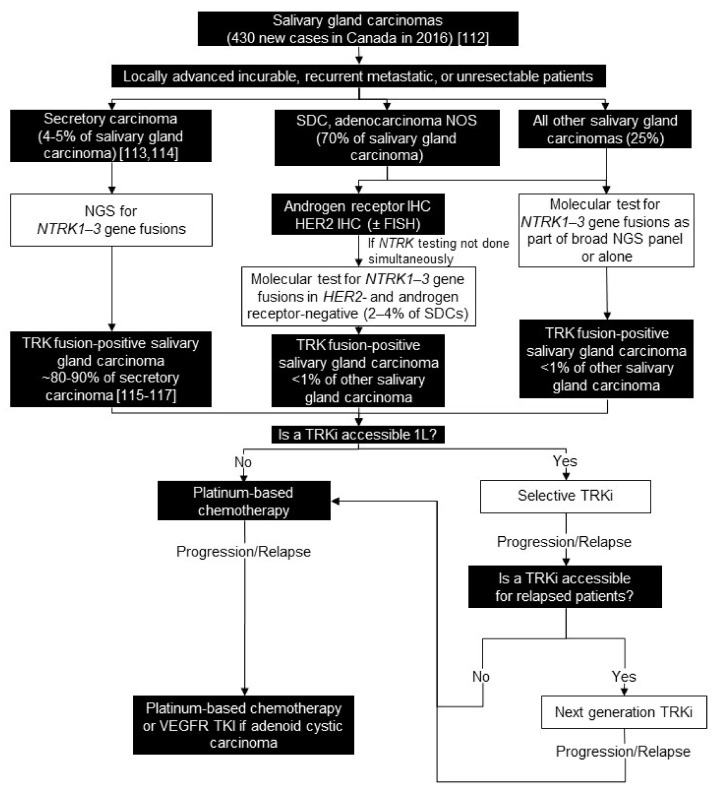
Biomarker testing and treatment for neurotrophic tyrosine receptor kinase (*NTRK)* gene fusions in salivary tumours. White boxes with black outlines represent either *NTRK* gene fusion testing or treatment with a TRKi. Black boxes with white text indicate all other steps that do not include either NTRK gene fusion testing or treatment with a TRKi. FISH = fluorescence in situ hybridization; L = line; *HER2* = erb-b2 receptor tyrosine kinase 2; IHC = immunohistochemistry; NGS = next generation sequencing; NOS = not otherwise specified; SDC = salivary ductal carcinoma; TRK = tyrosine receptor kinase; TRKi = TRK inhibitor; VEGFR TKI = vascular endothelial growth factor receptor tyrosine kinase inhibitor. Canadian Cancer Society [112]; Luk et al. 2015 [113]; Majewska et al. 2015 [114]; Black et al. 2019 [115]; Ito et al. 2015 [116]; Skalova et al. 2018 [117].

If the histology is unclear, we recommend routine broad molecular testing by NGS for targetable mutations, which includes *NTRK1-3* gene fusions. We estimate there are 5–20 patients with salivary gland carcinoma at each major centre in Canada/year and suggest for this number of patients a pan-TRK IHC screen is not recommended; however, it could be considered to conserve resources (Figure 5).

### 7.3. Treatment Consensus

We consider systemic treatment, such as chemotherapy, in patients with significant, symptomatic, systemic disease despite local therapy. We recommend first-line treatment with a selective TRK inhibitor for all patients with TRK fusion-positive salivary gland carcinoma (Figure 5). There are no systemic treatment options that have been shown to improve OS in metastatic patients. Some promising data with axitinib has emerged, but the OS data has yet to mature [124]. Thus, there is an urgent need for effective therapies, and impressive efficacy has been demonstrated with TRK inhibitors in these patients [16,17].

## 8. Tumour-Agnostic

### 8.1. Background

In adult tumours, secretory carcinoma of the salivary gland and the breast have a high frequency (>80%) of *NTRK* gene fusions, most often the *ETV6-NTRK3* gene fusion [6,7,31,125], and a subset have *ETV6-RET* fusions [117]. Papillary thyroid carcinomas have an intermediate frequency (5–25%) of *NTRK* gene fusions, and most other tumour types have a low (<5%) frequency [1,31,125,126,127].

Rosen and colleagues used a database of ~26,000 prospectively sequenced patients to identify those with *NTRK* gene fusions and assess their outcomes [15]. In 76 patients with TRK fusion cancer, the median PFS on first-line therapy (excluding TRK inhibitors) was 9.6 months, and ORR was 46.7% [15]. This illustrates that TRK fusion cancer can respond to standard of care, but not with a high ORR [15]. In this analysis, only one of 12 patients responded to a checkpoint inhibitor [15]. There is also considerable variability in treatment response to current standards of care across histologies.

### 8.2. Testing Consensus

**Figure 6:**
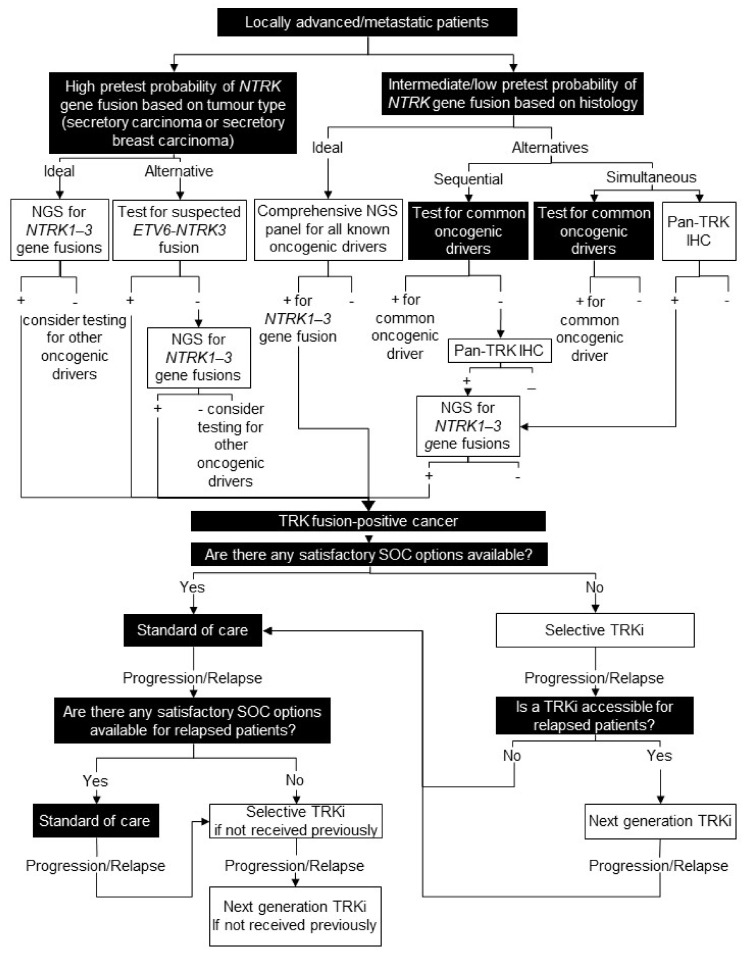
Biomarker testing and treatment for neurotrophic tyrosine receptor kinase (*NTRK)* gene fusions. White boxes with black outlines represent either *NTRK* gene fusion testing or treatment with a TRKi. Black boxes with white text indicate all other steps that do not include either NTRK gene fusion testing or treatment with a TRKi. *ETV6* = ETS variant transcription factor 6; IHC = immunohistochemistry; NGS = next generation sequencing; SOC = standard of care; TRK = tyrosine receptor kinase; TRKi = TRK inhibitor.

For secretory carcinoma of the salivary gland and the breast, since the fusion partners are well known and highly prevalent, a specific test (e.g., FISH, RT-PCR) for the *ETV6-NTRK3* gene fusion is an option [83]. Positive results would confirm the *ETV6-NTRK3* gene fusion, and patients with negative results should have access to an RNA-based NGS panel for broader *NTRK1-3* gene fusions. For tumour types with an intermediate or low probability of harbouring an *NTRK* gene fusion, ideally all metastatic/locally advanced patients would receive a comprehensive RNA-based NGS panel for all known oncogenic drivers, including *NTRK1-3* gene fusions. As this is not currently standard practice in Canada, we recommend either pan-TRK IHC testing simultaneous with funded testing for common oncogenic drivers, or pan-TRK IHC testing sequentially in wildtype tumours, because *NTRK* gene fusions are generally mutually exclusive with other common oncogenic drivers [15]. Confirmatory NGS or other molecular test is required for IHC-positive or equivocal samples (Figure 6).

### 8.3. Treatment Consensus

If there are standard of care treatment options that are considered satisfactory, we recommend exhausting these before treating a patient known to harbour an *NTRK* gene fusion with a TRK inhibitor, in accordance with the Health Canada-approved label. In areas of high unmet need, where none of the available options are considered satisfactory (e.g., surgery with high morbidity, excessive toxicity, or low response rates), it is reasonable to consider a TRK inhibitor as the first systemic treatment in patients with TRK fusion cancer (Figure 6).

## 9. Access to NTRK Gene Fusion Testing in Canada

Currently in Canada, access to *NTRK* gene fusion testing is limited, but is available through multiple channels and continues to improve. At the time of writing, *NTRK* testing is available via industry-sponsored programs, some government funded national programs, and direct to consumer programs as summarized in Table 1. Meanwhile, the ongoing CANTRK Ring Study aims to establish concordance at 17 sites across Canada for pan-TRK IHC and NGS testing for *NTRK* gene fusions [33].

It would be reasonable to consider assessing *NTRK* fusion status in all NGS assays, preferably RNA-based NGS panels, as the prevalence for *NTRK* fusions is low, but present in many tumour types.

## 10. Regulatory Landscape of TRK-Targeted Therapy in Canada and Elsewhere

The first tumour-agnostic agent approved by Health Canada was larotrectinib [19]. This represents a major paradigm shift in Canadian oncology. To confirm the real-world applicability of the Phase I/II results, the approval contained a requirement to provide post-market safety monitoring data.

Following the approval, the pan-Canadian Oncology Drug Review (pCODR) Expert Review Committee (pERC) did not recommend public payer reimbursement of larotrectinib in October 2019, due to uncertain net clinical benefit [128]. Also in October 2019, the Institut national d’excellence en santé et en services sociaux (INESSS) did not recommend public payer reimbursement [129]. In 2019, entrectinib was submitted to pCODR and INESSS for review for patients with *NTRK* fusion-positive solid tumours; however, as of September 2020, Hoffmann-La Roche Limited voluntarily withdrew the file from pCODR, and INESSS no longer listed the file as under review [130]. In contrast, in May 2020 for larotrectinib and June 2020 for entrectinib, the National Institute for Health and Care Excellence (NICE) in the United Kingdom recommended conditional coverage on its Cancer Drugs Fund [131,132]. Thus, when next reviewed by Canadian health technology agencies, a positive recommendation is possible for these targeted therapies.

As precision medicine evolves and new targets are discovered, smaller subgroups of patients characterized by their tumour’s specific molecular makeup are being identified. Phase III trials are not easy to conduct in these populations. Consequently, new standards are being developed to assess the utility of new treatments in these uncommon or rare molecular-defined cancers in a histology-agnostic manner.

## 11. Conclusions

Patients presenting with TRK fusion driven cancer represent an area of high unmet need. Often the currently available options for treatment offer low response rates and/or poor tolerability, and at relapse, options are limited to clinical trial or supportive care. In contrast, TRK inhibitors have demonstrated impressive response rates and good tolerability across tumour types in patients with TRK fusion cancer and should therefore be considered as a new therapeutic option. Currently, a major challenge is identifying TRK fusion cancer in a methodologically consistent way that is economically feasible in a public healthcare system.

Ideally, all patients could be tested with a comprehensive NGS panel for all possible actionable oncogenic alterations at diagnosis. With a plethora of new targets being identified, it is envisioned that in future, the massively parallel analysis of large NGS panels will become routine standard of care for all solid tumours. However, since this is not currently feasible in Canada, strategies for *NTRK*-positive patient enrichment and *NTRK* screening must be deployed. In current circumstances, a pan-TRK IHC screen is recommended in patient populations as described who are wildtype for other known oncogenic drivers. Patients whose tumours test positive or are inconclusive by IHC should then receive confirmatory NGS. In tumours with a canonical *ETV6-NTRK3* gene fusion, such as secretory carcinoma of the salivary gland and of the breast, a FISH test can be used to identify positive cases. Cases which test negative by FISH could receive NGS for *NTRK1-3* or *ETV6* gene fusions to rule out other possibilities. We recommend a selective TRK inhibitor in all patients with TRK fusion cancer with no other satisfactory treatment options.

The wide variability in testing availability and processes across Canada is a limitation of this consensus. This consensus is intended to offer general principles and should be adapted according to the tumour histology and the testing methods/procedures available at each individual Canadian solid tumour biomarker lab, at the discretion of the pathologist and molecular lab director. 

Future study should focus on further characterizing patients with TRK fusion cancer with the aim to identify subpopulations more likely to harbour *NTRK* gene fusions and prioritize them for testing. Another potential area of study is further investigation into the sequencing of TRK inhibitors for patients with TRK fusion cancer. 

We believe that this consensus will assist Canadian healthcare professionals in identifying patients with TRK fusion cancer and treating these patients, who would otherwise have had no satisfactory options, with effective and well-tolerated TRK inhibitors.

## Figures and Tables

**Table 1:** *NTRK* gene fusions testing options in Canada.

**Government-funded**
NGS available at select institutions/centres Exactis’ Personalize my Treatment (exactis.ca/personalize-my-treatment/)
**Industry-sponsored**
• *Fast*TRK (fasttrk.ca)
**Private, direct to consumer (out of pocket cost for patient)**
Canexia Health (canexiahealth.com/) Oncohelix (oncohelix.org/)
